# A Study on the Effect of Flow Unsteadiness on the Yield of a Chemical Reaction in a T Micro-Reactor

**DOI:** 10.3390/mi12030242

**Published:** 2021-02-27

**Authors:** Alessandro Mariotti, Matteo Antognoli, Chiara Galletti, Roberto Mauri, Maria Vittoria Salvetti, Elisabetta Brunazzi

**Affiliations:** Dipartimento di Ingegneria Civile e Industriale, Università di Pisa, Largo Lazzarino 2, 56122 Pisa, Italy; alessandro.mariotti@unipi.it (A.M.); matteo.antognoli@phd.unipi.it (M.A.); roberto.mauri@unipi.it (R.M.); maria.vittoria.salvetti@ing.unipi.it (M.V.S.); elisabetta.brunazzi@unipi.it (E.B.)

**Keywords:** T-shaped micro-mixer, unsteady flow regimes, mixing degree, reaction yield, experiments and numerical simulations

## Abstract

Despite the very simple geometry and the laminar flow, T-shaped microreactors have been found to be characterized by different and complex steady and unsteady flow regimes, depending on the Reynolds number. In particular, flow unsteadiness modifies strongly the mixing process; however, little is known on how this change may affect the yield of a chemical reaction. In the present work, experiments and 3-dimensional numerical simulations are carried out jointly to analyze mixing and reaction in a T-shaped microreactor with the ultimate goal to investigate how flow unsteadiness affects the reaction yield. The onset of the unsteady asymmetric regime enhances the reaction yield by more than 30%; however, a strong decrease of the yield back to values typical of the vortex regime is observed when the flow undergoes a transition to the unsteady symmetric regime.

## 1. Introduction

The growing interest towards microreactors is triggered by the possibility to combine continuous flow operations with an unprecedented control of operating conditions. This unique feature opens the possibility to reduce reactants, wastes and energy consumption, enabling more sustainable processes. Indeed, it is well accepted that microfluidics represents the main route towards process intensification to improve the yield and the selectivity of chemical reactions while limiting their environmental impact [1]. It has been estimated that around 50% of the homogeneous reactions and 30% of heterogeneous reactions, which are encountered in pharmaceutical and fine chemistry processes, may benefit of microfluidics [2,3,4]. Moreover, microreactors give the chance to explore new and greener synthesis routes, as the huge surface-to-volume ratio ensures very high heat transfer rates, allowing to safely deal with very exothermic or too dangerous reactions [5].

Despite the above fascinating characteristics, the actual application of microreactors has been hindered so far; the main reason is the difficulty to efficiently mix reactants [6]. Indeed mixing occurs solely through diffusion due to the laminar regime, which is determined by the tiny dimensions. Hence many efforts have been devoted at enhancing mixing through active or passive techniques. The latter methods are appealing as they do not need any external energy source and are more robust than active ones. These passive microreactors rely on a special geometrical arrangement of the inlet and mixing channels aimed at promoting the mixing of the liquid streams through convection [7].

The simplest example of a passive microreactor is the T-junction; despite such a basic configuration, its fluid dynamics is very intricate and exhibits different flow regimes, depending on the Reynolds number, i.e., Re [8]. For very low Reynolds numbers, the two inlet streams entering the T-shaped microreactor remain completely segregated in the mixing channel (segregated regime) with low mixing due to diffusion. With increasing Re, 3D U-shaped vortical structures stem from the confluence between the inlet streams and generate a double pair of counter-rotating vortical legs in the mixing channel (steady symmetric or vortex regime). Further augmenting the flow rate, the top part of the U-shaped vortical structures tilts and, as a consequence, one of the two legs becomes more intense than the other, which soon disappears in the channel (steady asymmetric or engulfment regime). This latter flow regime ensures a remarkable increase in the degree of mixing, because of the associated loss of symmetry. Then, the flow becomes unsteady and time-periodic, although being still in the laminar regime, preserving an intense asymmetry (unsteady asymmetric regime). The flow oscillations further push mixing, resulting in values of the degree of mixing that are even 30–50% larger than those of the engulfment regime [9,10,11]. However and unexpectedly, a further increase of Re triggers a flow, which, although still time-periodic, exhibits a large degree of symmetry, thus strongly hampering mixing (unsteady symmetric regime). Finally, the flow becomes chaotic.

The literature on the above flow regimes occurring in T-shaped microreactors is impressively extensive with both experimental and numerical investigations. It covers the effect of operating conditions [12], the insight on mechanisms promoting flow regime transitions [13], the effect of channel aspect ratios [14,15,16], the effect of the inclination [17,18,19,20] and position of the inlet channels [21,22,23], the presence of obstacles [24] as well as influence of fluid properties [10,25,26,27]. Some works investigate the influence of inlet flow conditions, e.g., not fully developed flow [28] or swirled flow [29]) on flow regimes. Besides, a dimensionless number taking into account the T-mixer geometry, has been proposed in the literature to identify the onset of the different steady flow regimes [30]. A comprehensive review on this topic has been recently provided by Camarri et al. [31]. However, in most of the above investigations the focus is on how flow regimes affect mixing rather than the progress of a chemical reaction.

In our recent works [32,33], we attempted to fill this gap by providing an insight on the role that steady flow regimes play on the performance of a chemical reaction in a T-shaped microreactor. More specifically, we considered different kinetic constants to comprehend the interplay between flow and kinetics. We highlighted different behaviors depending on the flow regime: in the segregated regime, the yield η of a chemical reaction depends solely on the flow to chemical time-scale ratio, i.e., η=f(Da), where Da is the Damköhler number, while in the vortex and engulfment regimes, an explicit dependency on the kinetic constant $k_{r}$ was observed with $\eta =f(k_{r},Da)$.

The present work, aims at extending the above investigation to unsteady flows. Firstly, we want to assess the presence of unsteady motions even for reactive fluids. Secondly, we aim at analyzing the impact of these motions on the progress of a chemical reaction, comparing the behavior to that of the steady regimes. To this purpose, experiments and numerical simulations are carried out jointly.

## 2. Experimental Setup

**Figure 1:**
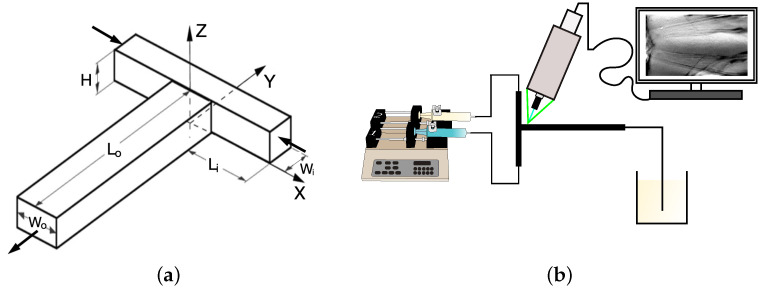
(**a**) Sketch of the T-reactor geometry and reference system; (**b**) experimental set-up.

The T-shaped microreactor and the experimental setup are the same as in the work of [32], therefore only a few details are briefly recalled herein. The inlet channels of the microreactor are characterized by a square cross-section, with width $W_{i}=H=$ 1 mm, while the mixing channel has a rectangular cross-section with aspect ratio 2:1, i.e., $W_{o}=2H=2$ mm. Hence, the hydraulic diameter of the mixing channel is d=4H/3. In the following text, coordinates will be made non-dimensional by using the hydraulic diameter, i.e., X=x/d, Y=y/d, and Z=z/d. The inlet channels length is equal $L_{i}=30d$, so the flow is fully developed at the T-junction, whereas the mixing channel length is equal to $L_{o}=45d$ and assures the complete evolution of vortical structures. The geometry of the reactor and the reference system of coordinates are provided in Figure 1a, while the experimental setup is shown in Figure 1b.

The test reaction is the reduction at ambient temperature of methylene blue (MB^+^), a water-soluble dye molecule, to the corresponding colorless leuco compound (LMB^+^) by adding ascorbic acid (AsA). An additional reaction product is the dehydroascorbic acid (DA). Such reaction is catalyzed by hydrogen chloride (HCl).

$${\mathrm{MB}}^{+}+\mathrm{AsA}\to {\mathrm{LMB}}^{+}+\mathrm{DA}$$

The aqueous solution of ${\mathrm{MB}}^{+}$ and HCl enters the reactor from one inlet while the aqueous solution of AsA is fed into the other inlet. The two reactant solutions were prepared as follows: 17 mg/L of ${\mathrm{MB}}^{+}$ powder (by Sigma Aldrich) are dissolved in aqueous solutions of 1 mol/L of HCl (by Sigma Aldrich), thus obtaining a concentration of ${\mathrm{MB}}^{+}$ equal to [${\mathrm{MB}}^{+}$] $=5.31\times 10^{-5}$ mol/L; as for the ascorbic acid solution, 300 mg/L of L-Ascorbic Acid (Ultrafine Vitamin C-powder by Cutatonic^®^) are dissolved in deionized water, to obtain an ascorbic acid concentration equal to [AsA] =1.7 mol/L. A syringe pump (KD Scientific), equipped with two Becton Dickson plastic syringes of 60 mL, was used to introduce the two streams at the same flow rate, and thus equal inlet bulk velocity, *U*. The pump is stopped after each test and then started again imposing the new flow rate; this procedure is aimed at avoiding any hysteresis phenomena in the mixing and reaction process.

In case of an excess of ascorbic acid, i.e., when [AsA] ≫ [${\mathrm{MB}}^{+}$] as in the present case, the above reaction has a pseudo-first order kinetic [34], that depends on the concentration of ${\mathrm{MB}}^{+}$ only with a kinetic constant, $k_{r}=10.71$ 1/s (see [32] for more details).

We selected this reaction because we can exploit the decolorization of the blue reactant to visualize the progress of the reaction at the confluence region and along the mixing channel, and thus study the effect of the flow regimes and mixing on the reaction yield. To this purpose, an upright microscope (Nikon, model Eclipse 80i), equipped with a magnifying lens of 4× (aperture *N.A.* = 0.13), a D-LH 12 V-100 W halogen lamp and a high-speed camera (Optomotive Velociraptor HS) with an up-mounted 0.7× lens, were used. Images were acquired with a resolution of 780 × 1504 pixels and an acquisition frequency of 450 frames/s. The image exposure time is significantly shorter than the one related to the frame rate, i.e., $\delta T=10^{-6}$ s. Each image collects a region of 1.70d×3.3d (the equivalent dimension of a pixel is 2.93 μm). Among the species involved in the reaction, ${\mathrm{MB}}^{+}$ is the only light-absorbing species. Therefore, the resulting intensity of the light across the specimen that is captured by the camera depends only on the MB^+^ concentration.

The Lambert-Beer’s law was used to convert light intensity into normalized depth-averaged ${\mathrm{MB}}^{+}$ concentration for each pixel of the image and was implemented in an offline procedure enabling the post processing of the flow visualizations. Additional details on calibration, image post-processing, and experimental setup are provided in [35].

## 3. Numerical Setup

The flow can be described by unsteady Navier-Stokes equations and transport/reaction equations for the chemical species ${\mathrm{MB}}^{+}$, ${\mathrm{LMB}}^{+}$, AsA, HCl and DA. The energy equation is not resolved as thermal effects can be neglected. The non-dimensional form of the equation is:

(1) $$\frac{\partial \hat{\rho }}{\partial \theta }+\nabla \cdot (\hat{\rho }u)=0,$$

(2) $$\hat{\rho }(\frac{\partial u}{\partial \theta }+u\cdot \nabla u)=-\nabla p+\frac{1}{Re}\nabla \cdot [\hat{\mu }(\nabla u+\nabla u^{T})]+Ri(\hat{\rho }-1)\hat{g},$$

(3) $$\hat{\rho }(\frac{\partial \phi_{k}}{\partial \theta }+u\cdot \nabla \phi_{k})=\frac{1}{Pe}\nabla \cdot (\hat{\rho }\hat{D_{k}}\nabla \phi_{k})+\frac{d{\dot{\omega }}_{k}}{\rho_{0}U},$$

where the lengths are normalized with the mixing channel hydraulic diameter *d* and velocities with the inlet bulk velocity *U*. θ is the non-dimensional time, i.e., $\theta =\frac{tU}{d}$. In the above equations, u represents the velocity vector, *p* is the modified non-dimensional pressure, i.e.,

(4) $$p=\left(P-\rho_{0}gZ\right)/\rho_{0}U^{2}$$

where *P* is the pressure, and *g* the gravity acceleration, while $\hat{g}$ is the non-dimensional gravity, i.e., $\hat{g}=\frac{g}{g}$. $\hat{\rho }=\rho /\rho_{0}$ and $\hat{\mu }=\mu /\mu_{0}$ are non-dimensional density and viscosity, respectively, referred to a convenient reference state, i.e., $\mu_{0}$ and $\rho_{0}$, which in our case corresponds to pure water at 20 °C. $\phi_{k}$ represents the mass fraction of the *k*-th chemical species while ${\dot{\omega }}_{k}$ is its rate of production or consumption due to chemical reactions, which, for instance is:

(5) $${\dot{\omega }}_{{\mathrm{MB}}^{+}}=-k_{r}\rho \phi_{{\mathrm{MB}}^{+}}$$

for the methylene blue. $\hat{D_{k}}=D/D_{0}$ is the non-dimensional diffusivity referred to the water self-diffusivity $D_{0}$.

The characteristic non-dimensional numbers are:

- the Reynolds number
  (6) $$Re=\frac{\rho_{0}Ud}{\mu_{0}}$$
- the Richardson number
  (7) $$Ri=\frac{gd\;\Delta \rho }{\rho_{0}U^{2}}$$
- the Peclet number
  (8) $$Pe=\frac{Ud}{D_{0}}$$

where $\nu_{0}=\mu_{0}/\rho_{0}$ is the kinematic viscosity of water, while Δρ is the density difference between the two inlet fluids.

Both fluid density and viscosity depends on the ascorbic acid content $\phi_{AsA}$ and hence, the correlations

(9) $$\mu =\mu_{o}f\left(\phi_{AsA}\right)$$

(10) $$\rho =\rho_{o}f\left(\phi_{AsA}\right)$$

from [36] are implemented in the model, see for details [32]. The fluid stream carrying AsA is 1.17 denser, at the inlet, than the other stream; indeed some stratification was observed to take place at Re<60 for the given size of our microreactor [32]. Despite the fact that stratification is therefore negligible in the range of Re for which unsteady flows occur, we retained the full formulation of the governing equations, including the term related to gravity.

The boundary conditions consist of: uniform velocity and concentration of the reactants at the entrance of the inlet channels; no-slip velocity at the channel walls and pressure outlet condition with ambient pressure at the outlet.

The transient solver of the finite volume code ANSYS Fluent v.19 [37], was utilized to solve the above equations. Time advancement was handled with a second order implicit advancement method, by using a time step corresponding to a CFL number ≅5, while a second order upwind interpolation scheme was used for spatial discretization. The PISO algorithm was employed for the pressure–velocity coupling. Such numerical setup has been successfully bench-marked against a massive parallel spectral element code in [38] where details on the grid independence study are also given. The computational grid is fully structured with 4.7 M cells; the cells are cubical at the confluence region, while they elongate along the inlet and the outlet channels.

## 4. Results

**Figure 2:**
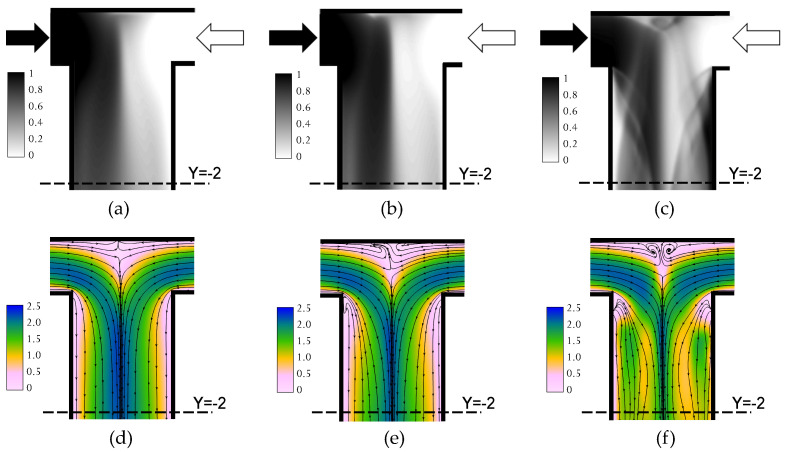
Depth-averaged ${\mathrm{MB}}^{+}$ concentration from numerical simulations in: (**a**) the segregated regime, (**b**) the vortex regime, and (**c**) the engulfment regime. Non-dimensional velocity magnitude and flow streamlines in the horizontal mid-plane in: (**d**) the segregated regime, (**e**) the vortex regime, and (**f**) the engulfment regime.

Figure 2 summarizes the steady flow regimes, namely segregated, vortex and engulfment regimes, which have been already characterized in Mariotti et al. [32]. The images in Figure 2a–c represent the depth-averaged ${\mathrm{MB}}^{+}$ concentration, estimated from the numerical simulations, in the confluence region. Such concentration is reported in non-dimensional form, being 1 the ${\mathrm{MB}}^{+}$ concentration in the inlet.

The agreement with the experimental flow visualizations has already been discussed in the same paper. Clearly, the engulfment regime (Figure 2c at Re=230) breaks the flow symmetries, thus strongly enhancing mixing with respect to the segregated (Figure 2a at Re=60) and vortex regimes (Figure 2b at Re=100), which both exhibit a prevailing dark region, corresponding to the methylene blue aqueous solution, on the left and a light region, corresponding to the aqueous solution of ascorbic acid, on the right. The non-dimensional velocity magnitude, i.e., normalized with the bulk velocity, in the horizontal mid-plane is reported in Figure 2d–f along with the flow streamlines. We observe that in both the segregated and vortex regimes the inlet streams flow side by side in the channel; however, a recirculation region is visible at the top of the microreactor. This recirculation region is more intense in the engulfment regime, where the fingerprint of the top part of the U-shaped vortical structures, which are tilted, can be well discerned from the streamlines.

**Figure 3:**
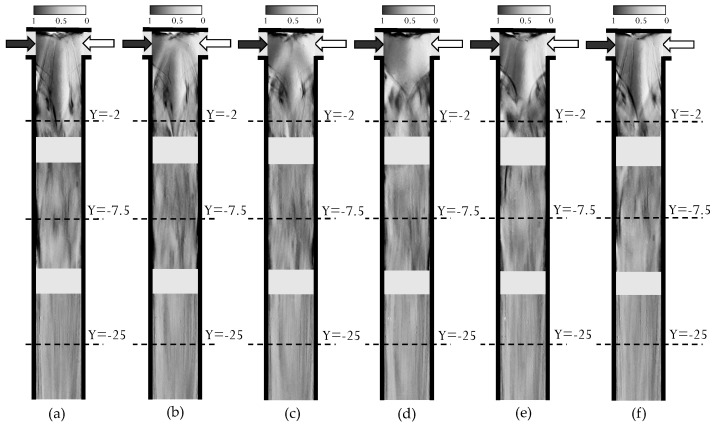
Experimental depth-averaged ${\mathrm{MB}}^{+}$ concentration at Re=320, for instants: (**a**) t/τ=0, (**b**) t/τ=0.17, (**c**) t/τ=0.33, (**d**) t/τ=0.5, (**e**) t/τ=0.67, (**f**) t/τ=0.83.

The experimental visualization of the time-periodic flow occurring at Re=320 is reported in Figure 3, which shows six different instants within a temporal cycle, as well as in the Videos S1 and S2. For each experimental test, images were acquired firstly in the confluence region, at −2.55<Y<0.75, then in the region at −9.15<Y<−5.85 and further along the mixing channel −26.65<Y<−23.35. Indeed, the flow visualization of the entire mixing channel was not undertaken as it would lead to a poor resolution because of the large length-to-width ratio of the channel. Therefore, we remark that the flow visualizations in the different regions, illustrated in Figure 3, are not synchronized with each other.

The temporal evolution of the ${\mathrm{MB}}^{+}$ concentration, confirms the occurrence and features of the asymmetric unsteady regime, that has been previously observed in literature with non-reactive systems [39]). More specifically, at the T-junction (top series of images) the initial flow configuration resembles the one of the engulfment regime. Here the top part of the U-shaped vortical structures is tilted, but during the temporal evolution, the tilting angle increases while the vortical structures approach each other, merge and annihilate. Simultaneously, the co-rotating vortical structures in the mixing channel generate a blob of vorticity (see top of Figure 3c), which is convected along the mixing channel (see top of Figure 3d,e). Moving downstream in the mixing channel (middle series of image), we notice a high degree of mixing, being the color of the images rather uniform, however exhibiting some signatures of the unsteady flow. Such signatures attenuate while moving further downstream (bottom series).

**Figure 4:**
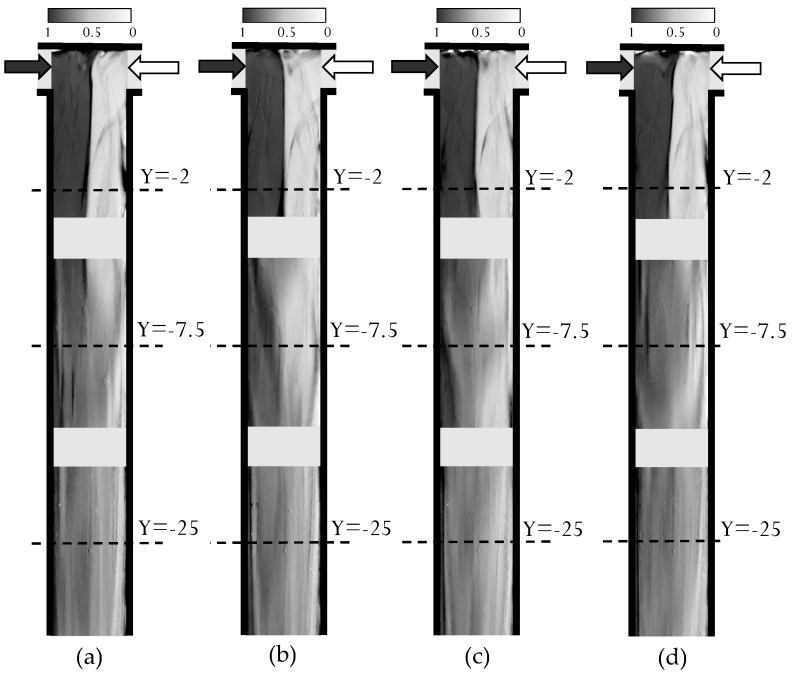
Experimental depth-averaged ${\mathrm{MB}}^{+}$ concentration at Re=650 for instants: (**a**) t/τ=0, (**b**) t/τ=0.25, (**c**) t/τ=0.50, (**d**) t/τ=0.75.

Similar experimental snapshots are shown in Figure 4 for Re=650. The related video is provided in the Supplementary Material. Here we observe how at the confluence the flow preserves a large degree of symmetry, resembling the vortex regime shown in Figure 2b, except for the dynamic feature. Hence, this symmetric unsteady regime, which has been already observed when feeding the same fluid, i.e., water, to the microreactor, occurs also in the present case involving fluids with different density and viscosity. We notice from the ${\mathrm{MB}}^{+}$ concentration maps just small oscillations of the contact region. Mixing is clearly hampered with respect to the previous regime, despite the fact that the Re is almost doubled. Such segregation persists further down in the mixing channel, the bottom series of images still showing dark and white signatures of the inlet streams on the left and right, respectively.

**Figure 5:**
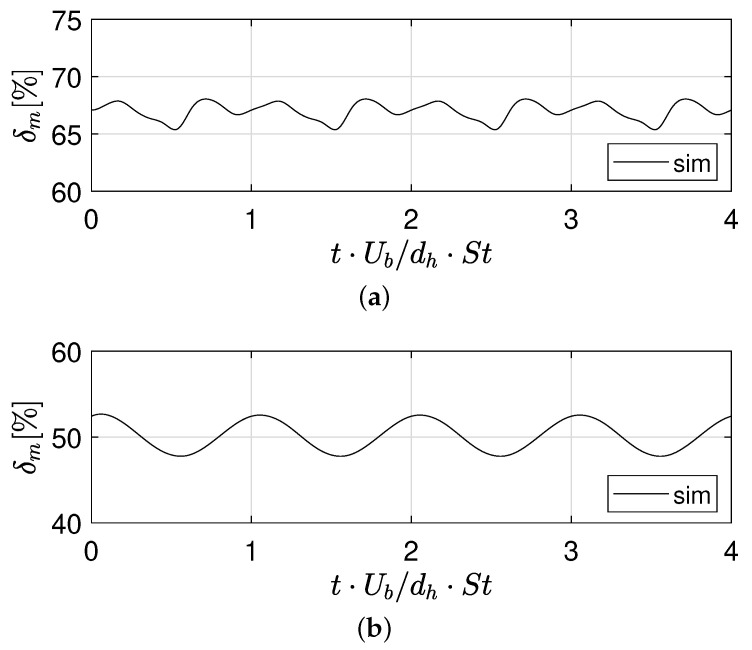
Numerical time signal of the mixing degree at Y=−25 for (**a**) Re=320 and (**b**) Re=650.

The corresponding degree of mixing at Y=−25 is reported in Figure 5 for the two Reynolds numbers at different instants, which are made non-dimensional with respect to the cycle period, i.e., $\tau =\frac{d}{U\cdot St}$ with St being the Strouhal number. At Re=320 we estimated St=0.253 from the numerical simulations, while a slightly lower St=0.228 was determined for Re=650. The experimental values of the Strouhal number were St=0.246 at Re=320 and St=0.224 at Re=650, hence showing a very satisfactory agreement with numerical ones.

The degree of mixing, $\delta_{m}$ is defined as in [26,32,38], i.e.,

(11) $$\delta_{m}=1-\frac{\sigma_{b}}{\sigma_{max}},$$

where $\sigma_{b}$ is the standard deviation of the volumetric dye flow and $\sigma_{max}$ is the maximum value of $\sigma_{b}$ obtained for completely segregated streams. Thus, $\delta_{m}$ varies between $\delta_{m}=0$, indicating a completely segregated flow, and $\delta_{m}=1$, corresponding to a perfectly mixed flow. In our case the dye is HCl, as this component does not participate in the chemical reaction and hence acts as a passive tracer.

For both Re, the mixing degree fluctuates being affected by the flow dynamics. In the unsteady asymmetric regime, i.e., at Re=320 we notice how the signal exhibits a double peak (see Figure 5a); one peak can be related to the passage of the vorticity blob, while the other to the instant when the vortical legs in the channel are strongest. In this regime, the averaged $\delta_{m}$ at Y=−25 is $\delta_{m}=65.9\pm 1.6\%$. In the unsteady symmetric regime, i.e., at Re=650, the dynamics is simpler with a nearly sinusoidal shape of the $\delta_{m}$ signal (see Figure 5b). The degree of mixing at Y=−25 is $\delta_{m}=50.1\pm 2.1\%$ as reported also in Table 1.

**Figure 6:**
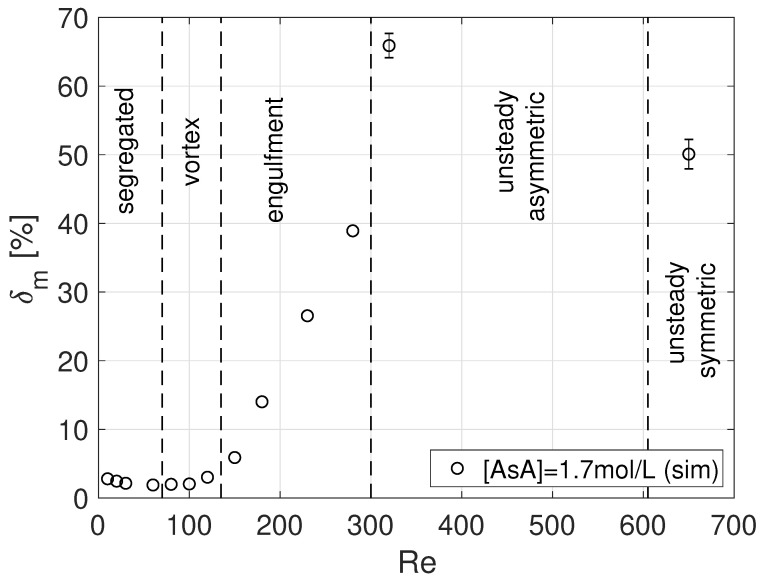
Mixing degree at Y=−25 as a function of Reynolds number for steady and unsteady regimes.

The above $\delta_{m}$ values are compared to those of the steady regimes in Figure 6. We notice how the unsteady motions boost mixing as $\delta_{m}$ increases from about 40% at Re=280 (i.e., steady asymmetric regime) to 65.9% at Re=320 (i.e., unsteady asymmetric regime); however a further increase of Re towards the unsteady symmetric regime is detrimental.

**Figure 7:**
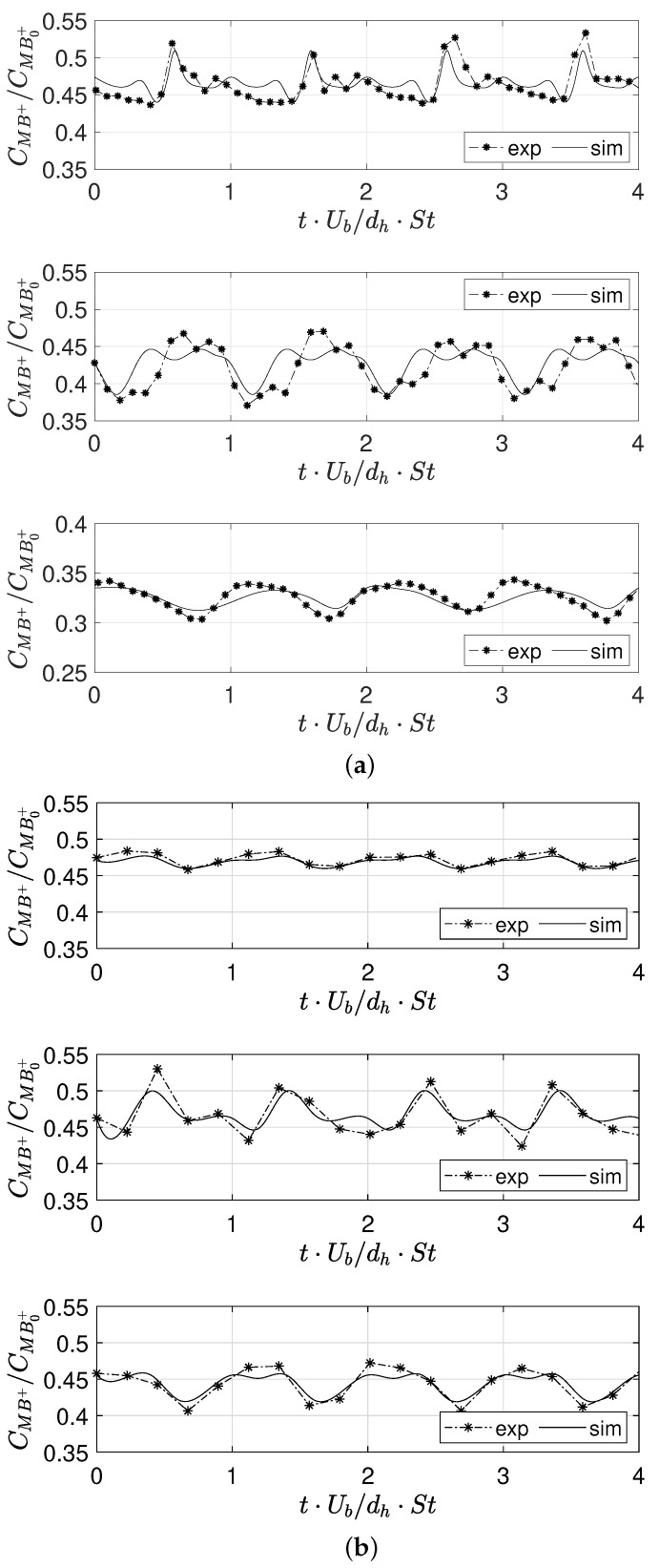
Experimental and numerical time signals of the ${\mathrm{MB}}^{+}$ concentration at for (**a**) Re=320 and (**b**) Re=650 at cross- sections (from top to bottom): Y=−2, Y=−7.5, and Y=−25.

Figure 7 compares experimental and numerical temporal signals of the average ${\mathrm{MB}}^{+}$ concentration, which is made non-dimensional with respect to the inlet ${\mathrm{MB}}^{+}$ concentration, at the Y=−2, Y=−7.5 and Y=−25 cross-sections (from top to bottom) and for Re=320 (Figure 7a) and Re=650 (Figure 7b).

We can notice how the ${\mathrm{MB}}^{+}$ decreases moving along the channel as the reaction proceeds and how all signals show the fingerprint of the dynamic behavior which affects the entire mixing channel. The good agreement between experiments and numerical simulations confirms that the present numerical setup is able to well capture the evolution of the chemical reaction.

**Figure 8:**
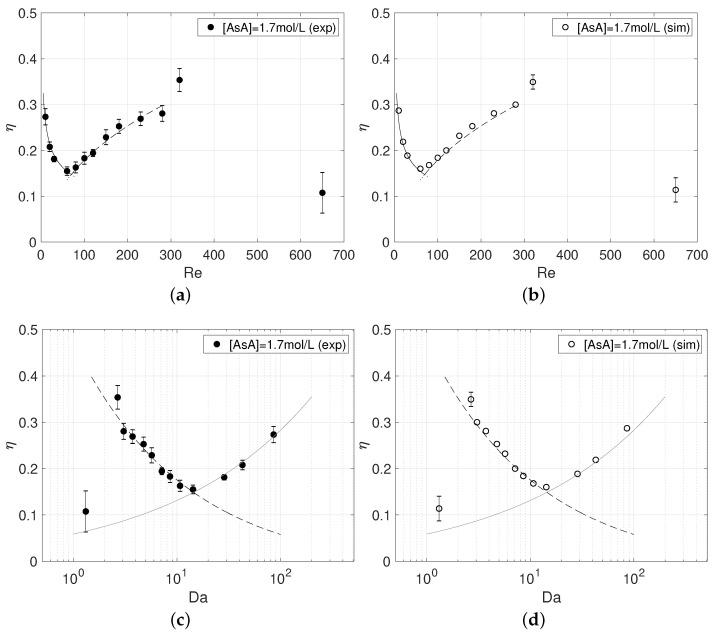
Reaction yield at Y=−25 in experiments (**a**,**c**) and numerical simulations (**b**,**d**) as a function of Reynolds number (**a**,**b**) and Damköhler number (**c**,**d**).

The corresponding experimental and numerical reaction yields are reported in Figure 8a–d, respectively, along with data from the steady regimes, already discussed in [32]. The reaction yield is evaluated at the Y=−25 from the ${\mathrm{MB}}^{+}$ concentration as:

(12) $$\eta =1-2\frac{C_{BM^{+},Y=-25}}{C_{BM_{0}^{+}}}$$

with $C_{BM^{+},Y-25}$ being the ${\mathrm{MB}}^{+}$ concentration averaged on the cross-section. The lines refer to the scaling of the reaction yield with the Reynolds and Damköhler numbers suggested in the aforementioned paper for the different regimes, respectively, i.e.,:

- in the segregated regime (see solid line)
  (13) $$\eta \propto Da^{0.3}$$
- in the vortex and engulfment regimes (dashed line).
  (14) $$\eta \propto \frac{{\tilde{k_{r}}}^{0.1}}{Da^{0.4}}$$

Here the Damköhler number, representing the flow to chemical time-scale ratio, is

(15) $$Da=dk_{r}/U=\tilde{k_{r}}/Re$$

and $\tilde{k_{r}}$ is the non-dimensional kinetic constant, i.e.,

(16) $$\tilde{k_{r}}=k_{r}\frac{d^{2}}{\nu }$$

The agreement between the experimental and numerical yield is very satisfactory. We notice how the yield at Re=320 is enhanced considerably with respect to the value we would expect in case of a steady engulfment regime. In particular η≈28% at Re=280 while increases up to η≈35% at Re=320. More precisely, η=34.9±1.6% in the simulations and η=35.4±2.5% in experiments, thus further confirming the accuracy of the numerical setup. Such an increase of reaction yield is motivated by the periodic motions which strongly boost the mixing of reactants.

Instead, the yield drops significantly at Re=650 down to η≈11% (η=11.4±2.7% in simulations and η=10.7±4.4% in experiments), thus at values that are even lower than those of the segregated regime, i.e., η≈15% for Re=60. Indeed, despite the fact that the oscillations of the contact region in the symmetric unsteady regime improve mixing with respect to the segregated regime, the yield is penalized by the much lower residence time at Re=650. It is worth noting that Figure 8c,d indicate how this yield is somewhat larger than the one we would expect from the $\eta \propto Da^{0.3}$ relationship, i.e., based solely on the residence time, because of the periodic motions.

## 5. Conclusions

In the present work experiments and numerical simulations were used jointly to investigate the presence of unsteady flows in case of reactive system and analyze their impact on the reaction progress of a chemical reaction. Two different unsteady regimes, i.e., asymmetric and symmetric, were observed with the same features already described for non-reactive flows. The numerical simulations provided results which were closely in agreement with the experiments. We highlight that a grid adaption cannot be effectively implemented in transient simulations to improve the description of the mixing of reactants; however the unsteady regimes further promote convection and increase the mixing length, making the computational grid fine enough to capture mixing.

The unsteady motions strongly boost the progress of the chemical reaction, its yield increasing by more than 30% when moving from the steady flow at Re=280 to the unsteady flow at Re=320. Such reaction enhancement is higher than the one we would estimate by considering the relationship $\eta \propto \frac{{\tilde{k_{r}}}^{0.1}}{Da^{0.4}}$ developed for the steady regime. According to this relationship, if η≈28% at Re=280 in the engulfment regime we would expect η≈30% at Re=320, while the unsteady motions push the yield up to η=35%.

However, the onset of the unsteady symmetric regime at higher flow rates, has a detrimental effect on the reaction progress, with η≈11%, thus lower than the yields of the steady segregated regime. In fact, despite oscillations of the contact region enhance somewhat the mixing of reactants, the very low residence time hampers the reaction progress.

To our knowledge the present work provides the first evidence that unsteady flow regimes may be effectively exploited to improve significantly the yield of chemical reactions. Hence we believe that this information is useful for enabling process intensification in microreactors.

## Figures and Tables

**Table 1:** Numerical values of the mixing degree and reaction yield at Y=−25.

*Re*	$\delta_{m}\left[\%\right]$	$\eta \left[\%\right]$
320	65.9±1.6	34.9±1.6
650	50.1±2.1	11.4±2.7

## Supplementary Materials

The following videos are available online at https://www.mdpi.com/2072-666X/12/3/242/s1, Video S1: Experimental depth-averaged ${\mathrm{MB}}^{+}$ concentration at Re=320.; Video S2: Experimental depth-averaged ${\mathrm{MB}}^{+}$ concentration at Re=650.

## Abbreviations

The following symbols and abbreviations are used in this manuscript:

Δρ	density difference between the two inlet fluids, kg m${}^{-3}$
$\delta_{m}$	degree of mixing, -
δT	image exposure time, s
η	reaction yield, -
θ	non-dimensional time, -
μ	dynamic viscosity, kg m${}^{-1}$ s${}^{-1}$
$\hat{\mu }$	non-dimensional kinematic viscosity, -
$\mu_{0}$	dynamic viscosity of pure water at 20 °C, kg m${}^{-1}$ s${}^{-1}$
$\nu_{0}$	kinematic viscosity of pure water at 20 °C, m${}^{2}$ s${}^{-1}$
ρ	density, kg m${}^{-3}$
$\hat{\rho }$	non-dimensional density, -
$\rho_{0}$	density of pure water at 20 °C, kg m${}^{-3}$
$\sigma_{b}$	standard deviation of the volumetric dye flow, -
$\sigma_{max}$	maximum value of the standard deviation of the volumetric dye flow, -
τ	cycle period, s
$\phi_{k}$	mass fraction of the *k*-th chemical species, -
${\dot{\omega }}_{k}$	rate of production or consumption of $\phi_{k}$ due to chemical reactions, kg m${}^{-3}$ s${}^{-1}$
AsA	ascorbic acid
$C_{BM^{+}}$	methylene blue concentration, mol m${}^{-3}$
CFL	Courant–Friedrichs–Lewy number, -
*d*	mixing channel hydraulic diameter, m
D	diffusivity, m${}^{2}$ s${}^{-1}$
$\hat{D_{k}}$	non-dimensional diffusivity, -
$D_{0}$	water self-diffusivity, m${}^{2}$ s${}^{-1}$
Da	Damköhler number, -
DA	dehydroascorbic acid
*g*	gravity acceleration, m s${}^{-2}$
$\hat{g}$	non-dimensional gravity, -
*H*	channel height, m
HCl	hydrogen chloride
$k_{r}$	kinetic constant, s${}^{-1}$
$\tilde{k_{r}}$	non-dimensional kinetic constant, -
$L_{i}$	inlet channel length, m
${\mathrm{LMB}}^{+}$	leuco compound
$L_{o}$	mixing channel length, m
${\mathrm{MB}}^{+}$	methylene blue
*N.A.*	numeric aperture, -
*p*	modified non-dimensional pressure, -
*P*	pressure, Pa
Pe	Peclet number, -
PISO	Pressure implicit with splitting of operator algorithm
Re	Reynolds number, -
Ri	Richardson number, -
St	Strouhal number, -
*t*	time, s
*U*	bulk velocity, m s${}^{-1}$
u	velocity vector, m s${}^{-1}$
$W_{i}$	inlet channels width, m
$W_{o}$	mixing channel width, m
*x*	x-coordinate, m
*X*	non-dimensional x-coordinate, -
*y*	y-coordinate, m
*Y*	non-dimensional y-coordinate, -
*z*	z-coordinate, m
*Z*	non-dimensional z-coordinate, -
